# Androgens alter the heterogeneity of small extracellular vesicles and the small RNA cargo in prostate cancer

**DOI:** 10.1002/jev2.12136

**Published:** 2021-08-18

**Authors:** Elena S. Martens‐Uzunova, Gina D. Kusuma, Stefania Crucitta, Hong Kiat Lim, Crystal Cooper, James E. Riches, Arun Azad, Takahiro Ochiya, Glen M. Boyle, Melissa C. Southey, Marzia Del Re, Rebecca Lim, Grant A. Ramm, Guido W. Jenster, Carolina Soekmadji

**Affiliations:** ^1^ Department of Urology, Erasmus MC, Cancer Institute University Medical Centre Rotterdam Rotterdam The Netherlands; ^2^ The Ritchie Centre, Hudson Institute of Medical Research Clayton Victoria Australia; ^3^ Department of Obstetrics and Gynaecology Monash University Clayton Victoria Australia; ^4^ Unit of Clinical Pharmacology and Pharmacogenetics, Department of Clinical and Experimental Medicine University Hospital of Pisa Pisa Italy; ^5^ Department of Cell and Molecular Biology QIMR Berghofer Medical Research Institute Brisbane Australia; ^6^ Central Analytical Research Facility Institute for Future Environments Queensland University of Technology Brisbane Australia; ^7^ Sir Peter MacCallum Department of Oncology University of Melbourne Parkville Victoria Australia; ^8^ Department of Medical Oncology Peter MacCallum Cancer Centre Melbourne Australia; ^9^ Institute of Medical Science Tokyo Medical University Tokyo Japan; ^10^ School of Biomedical Sciences, Faculty of Medicine University of Queensland Brisbane Australia; ^11^ Genetic Epidemiology Laboratory, Department of Pathology The University of Melbourne Melbourne Australia

**Keywords:** androgen, androgen receptor, enzalutamide, exosomes, extracellular RNA, extracellular vesicles, hormone, microRNA, prostate cancer, small RNA, testosterone

## Abstract

Proliferation and survival of prostate cancer cells are driven by the androgen receptor (AR) upon binding to androgen steroid hormones. Manipulating the AR signalling axis is the focus for prostate cancer therapy; thus, it is crucial to understand the role of androgens and AR on extracellular vesicle (EV) secretion and cargo. In this study, we report that plasma‐derived circulating vesicles consisting of CD9 and double‐positive for CD9 and Prostate Specific Membrane Antigen (PSMA) are increased in patients with advanced metastatic prostate cancer, whereas double positives for CD9 and CD63 small extracellular vesicles (S‐EVs) are significantly higher in patients with localised prostate cancer. Androgen manipulation by dihydrotestosterone (DHT) and the clinical antagonist enzalutamide (ENZ) altered the heterogeneity and size of CD9 positive S‐EVs in AR expressing prostate cancer cells, while assessment of the total number and protein cargo of total S‐EVs was unaltered across different treatment groups. Furthermore, hormone stimulation caused strong and specific effects on the small RNA cargo of S‐EVs. A total of 543 small RNAs were found to be regulated by androgens including miR‐19‐3p and miR‐361‐5p. Analysis of S‐EVs heterogeneity and small RNA cargo may provide clinical utility for prostate cancer and be informative to understand further the mechanism of resistance to androgen targeted therapy in castration‐resistant prostate cancer.

## 1 INTRODUCTION

Prostate cancer is the fourth most common cancer worldwide, with 1.3 million men diagnosed and 3600 deaths recorded in 2018 (Bray et al., 2018). Nearly all prostate cancers exhibit androgen‐dependent growth, and as a result, androgen deprivation therapy (ADT) is the mainstay of treatment for advanced prostate cancer. ADT aims to inhibit tumour growth by preventing the activation of a nuclear transcription factor, the androgen receptor (AR). The vast majority of men respond initially to ADT, but tumour progression is inevitable and indicated by a rising prostate‐specific antigen (PSA) level, new lesions, or progressive symptoms. This lethal phenotype is referred to as castration‐resistant prostate cancer (CRPC) but remains driven by persistent AR signalling (Watson et al., 2010). As a result, the AR axis remains a critical therapeutic target in patients with metastatic CRPC. Some factors have been reported to contribute towards the development of CRPC such as those mediated by AR amplification, expression of AR splice variants, AR mutations, extra‐gonadal androgen synthesis, enhanced activity of AR co‐regulators and upregulation of signalling cascades that further activate the AR pathway (Wyatt & Gleave, 2015).

Extracellular vesicles (EVs) are secreted by cells into the extracellular milieu and systemic circulation. EVs are secreted by virtually all cells and contain cellular component encapsulated by lipid membranes. EVs can deliver multiple bioactive components, including proteins, lipids, metabolites and nucleic acids, to neighbouring or distant cells. In so doing, EVs have been shown to be critical to cellular homeostasis, as well as disease pathobiology. Several studies have shown that EVs can be secreted by cells in response to external stimuli such as hormones (Soekmadji et al., 2016), drugs (Samuel et al., 2017), calcium (Savina et al., 2003), or a certain classes of lipids such as ceramides (Elsherbini & Bieberich, 2018). Our work has investigated the effect of the most potent male androgen, dihydrotestosterone (DHT) and the clinical non‐steroidal AR antagonist used to treat CRPC, enzalutamide (ENZ), on the protein cargo of small EVs (S‐EVs, 30–150 nm in diameter) (Merseburger et al., 2015; Soekmadji et al., 2016). DHT is the most potent male androgen that activates AR‐mediated signalling and affects mRNA and protein expression of target genes that regulate proliferation and survival. Secreted S‐EVs can exacerbate the proliferation of prostate cancer cells in androgen deprived condition (Soekmadji et al., 2016). We demonstrated that DHT increases the abundance of CD9 in the isolated total S‐EVs from prostate cancer cells (Soekmadji et al., 2016; Soekmadji et al., 2017). However, the effect of DHT on the heterogeneity of S‐EVs and other S‐EVs cargo components is unknown.

In the past few years, technologies have emerged and been adapted for EV analysis, particularly those that enable analysis of single EV. These technologies provide the possibility to characterise EVs based on transmembrane proteins, such as the most abundant tetraspanins. Kowal et al. demonstrated that isolation of EVs from dendritic cells using tetraspanin specific antibodies resulted in distinct protein cargo from S‐EV subpopulations (Kowal et al., 2016). Further, a live imaging technique has also demonstrated the difference between CD9 positive (CD9+) and CD63 positive (CD63+) EVs, where CD63+ EVs can be induced by stimulation of the histamine H1 receptor in HeLa cells. In contrast, CD9+ EVs are unaffected (Verweij et al., 2018). It has been reported in previous independent studies that the EV markers are expressed on discrete subcellular localisations, and that they also have specific biological functions (Soekmadji et al., 2013).

In prostate cancer, evidence strongly suggests that small RNAs such as micro RNAs (miRNAs) are involved in cancer progression to CRPC. MiRNAs alter the activity of a transcription factor by incorporating into the RNA‐induced silencing complex (RISC), allowing them to bind to the messenger RNAs (mRNAs) of target genes resulting in the decreased expression of the corresponding proteins through translational repression, mRNA cleavage or mRNA destabilisation (Carrington, 2003; Lim et al., 2005; Meister, 2007; Song, 2004). In a prostate cancer study, Östling et al. (2011) screened ∼1000 miRNAs and demonstrated that 71 unique miRNAs were shown to influence AR expression. Subsequent reporter assays identified 13 miRNAs that can directly bind to the AR mRNA and regulate AR protein expression levels (Östling et al., 2011). Others have investigated the effect of androgens on miRNA expression levels, such as on miR‐221/‐222 as reported by Gui et al. (2017). It has also been shown that androgens alter the expression of miR‐125b, which leads to the up‐regulation of the proapoptotic regulator Bak1 (Shi et al., 2007), potentially contributing to prostate cancer survival. MiRNAs have been demonstrated to exert their function outside of their cell of origin via EVs in cancer and other diseases (Blandford et al., 2018; Jurj et al., 2020; Melo et al., 2014; Skog et al., 2008; Valadi et al., 2007).

This study reports the heterogeneity of circulating S‐EVs in plasma from patients with benign prostate hyperplasia, localised prostate cancer and advanced metastatic prostate cancer, and those secreted by well‐described prostate cancer cell lines under androgen manipulation. While the expression and subcellular localisation of tetraspanin EV markers CD9 and CD63 are distinct in prostate cancer (Soekmadji et al., 2016), the effect of androgens such as DHT and ENZ on the S‐EVs profile is unclear. We tested the effect of androgens on the composition of S‐EVs subpopulations secreted by AR expressing prostate cancer cells. We also investigated the effects of androgens on small RNA cargo in prostate cancer cells. Paired comparison of small RNA cargo from parental cells and isolated S‐EVs was performed to assess the potential role of S‐EVs on AR‐mediated signalling. We further discuss the identified S‐EVs miRNAs in the context of AR signalling axis in prostate cancer.

## 2 MATERIAL AND METHODS

### 2.1 Plasma collection

Plasma from men with benign prostate hyperplasia (BPH, *n* = 10) was obtained from the Victorian Cancer Biobank, Melbourne (Ethics Approval no. 06160C) after transurethral resection of the prostate (TURP). All patients were diagnosed as BPH as confirmed by histology of prostate tissue and had no history of other malignancies for the previous 5 years. Plasma samples from men with localized prostate cancer (LPCa, *n* = 11) were also obtained from Victorian Cancer Biobank before digital rectal exam (DRE) at the time of day most convenient to the patient. For inclusion in the study, PSA levels and prostate biopsy results had to meet or exceed the following clinic‐pathological criteria: PSA > 20 ng/ml; ISUP Grade group 3 (Gleason score 7 (4+3)), group 4 (Gleason Score 8) ‐or group 5 (Gleason Score 9–10); clinical stage > / = T2c. All patients were treatment naïve, had had no detectable metastatic disease and no history of other malignancies for the previous 5 years. Preparation of fresh plasma was performed in less than 2 h from the time of receipt. Nine ml of blood was collected in ethylenediaminetetraacetic acid (EDTA) tubes and centrifuged at 1200 *g* then 1800 *g*, each for 10 min at room temperature to eliminate debris and cell contaminant. Plasma samples were stored at −80°C until further analysis.

Plasma from men with metastatic CRPC was collected after written consent (advanced PCa, AdvPCa, *n* = 15). Patients were diagnosed with histologically confirmed prostate adenocarcinoma, progressive disease despite castrate levels of serum testosterone (< 500 ng/l). Patients have documented metastases by computed tomography or technetium‐99 bone scans, at least three increasing serum PSA values taken at least 2 weeks before the last value of at least 2.0 ng/ml, consistent with the Prostate Cancer Working Group‐2 guidelines. The study was approved by the Ethics Committee of Pisa University Hospital (Ethics Committee–University Hospital of Pisa, Ethics Approval no. 5382) and conducted following the principles of the Declaration of Helsinki. All patients gave their signed informed consent before blood collection. Six ml of blood were collected prior to treatment with anti‐androgens using EDTA tubes and centrifuged at 1900 *g* for 10 min at 4°C within 2 h after drawing. Plasma was stored at ‐80°C until analysis.

### 2.2 Cell culture

Androgen receptor (AR) positive prostate cancer cells LNCaP and AR negative PC3 cells were purchased from ATCC. C42B cells were obtained from MD Anderson Cancer Centre (USA). LNCaP cells are cell lines derived from epithelial prostate cancer and are among the most well‐characterised prostate cancer models to investigate AR signalling in prostate cancer. Cells were cultured in phenol red‐free RPMI 1640 (Invitrogen) supplemented with 5% foetal bovine serum (FBS; Thermo Scientific). AR positive cells were seeded at 5000 cells/cm^2^ in T‐75 or T‐175 Nunc flasks or plastic tissue culture dishes to assess the response to androgens. Cells were grown in RPMI + 5% FBS for 72 h before replacing the medium with 5% charcoal‐stripped serum (CSS; Sigma). The use of CSS as a medium to investigate the AR signalling axis in prostate cancer cells has been performed routinely for the past two decades. Treatment with androgens for AR positive cells was performed in RPMI + 5% vesicle‐depleted CSS with EtOH (vehicle) or 10 nM dihydrotestosterone (DHT, Cayman Chemical) with or without 10 μM enzalutamide (ENZ, Selleckchem) and cultured for 48 h. Assessment of S‐EVs from AR negative PC3 cells was performed on cells cultured in RPMI + 5% vesicle‐depleted FBS for 48 h. Conditioned media were collected, and cells were harvested for further analysis. S‐EVs‐depleted serum was prepared by ultracentrifugation of RPMI + 20% FBS or CSS at 100,000 *g* for 16 hours at 4°C, followed by filtration using a 0.22 μm vacuum filter (Invitrogen). All chemicals and reagents were obtained from Sigma unless indicated otherwise.

### 2.3 S‐EVs isolation

Small extracellular vesicles (S‐EVs) were isolated from freshly collected conditioned media by a series of differential centrifugation steps, as previously described (Théry et al., 2006). Briefly, conditioned media were collected and centrifuged at 2000 *g* for 20 min to pellet cells and dead cells and 10,000 *g* for 30 min to pellet cellular debris. The supernatants were then subjected to ultracentrifugation at 100,000 *g* for 2 h to pellet S‐EVs using SW32Ti rotor. Collected pellets were washed once in PBS at 100,000 *g* for 1.5 h. S‐EVs were then resuspended in PBS for further experiments and stored at ‐80°C until analysis. Protein content was measured using the BCA protein assay kit (Pierce).

### 2.4 Vesicle count, size measurement and assessment on the heterogeneity of S‐EVs

Plasma samples were thawed on ice and centrifuged at 1600 *g* for 15 min at 4°C to eliminate cells and cellular debris. Samples were then centrifuged at 10,000 *g* for 30 min at 4°C to eliminate large EVs (> 200 nm in diameter). Cell culture conditioned media were collected at the end of three independent experiments and centrifuged at 2000 *g* for 20 min at 4°C to eliminate dead cells and debris and stored at ‐80°C before analysis. The particle size, quantity and surface protein characteristics of EVs were analysed through single particle interferometric reflectance imaging sensing (SP‐IRIS) using the ExoView platform (Nanoview Biosciences). ExoView uses a multiplex microarray chip for the immune‐capture of commonly expressed EV tetraspanin proteins CD9, CD63, CD81. ExoView then analyses EVs using visible light interference for size measurements and fluorescence for protein profiling.

Conditioned media and plasma were diluted according to the manufacturer's protocol for the ExoView Human Tetraspanin kit (EV‐TETRA‐C) and ExoView Plasma Tetraspanin kit (EV‐TETRA‐P), respectively, coated with antibody spots against human CD9, CD63, CD81 as well as IgG as isotype controls in triplicates. Plasma kit also contains CD41a antibody as a platelet EV marker. Following overnight incubation in a 24‐well plate, chips were washed three times on an orbital shaker with Solution A. Then, the chips were incubated for 1 hour at RT with a cocktail of fluorescent antibodies, that is, 0.5 μg/ml anti‐CD9‐AF488, 1 μg/ml anti‐PSMA‐AF555, and 0.5 μg/ml anti‐CD63‐AF647. Detection of Prostate Specific Membrane Antigen (PSMA) on circulating plasma‐derived S‐EVs was performed using humanised J591 antibody (Dr Neil Bander, USA), labelled using Alexa Fluor 555 Protein Labeling Kit (A20174, Molecular Probes) according to the manufacturer's instructions.

The chips were washed once in Solution A, three times in Solution B, once in deionized water and dried. Image and data acquisition was performed with the ExoView R100 reader using the nScan 2.8.10 acquisition software (NanoView Biosciences). Data analysis was performed with sizing thresholds set to 50 to 200 nm diameter and analysed using NanoViewer 2.8.10 (NanoView Biosciences).

The mean (+/‐ SEM) of the fluorescence intensity of S‐EVs was determined by subtracting the number of particles with those captured with negative control IgG captured antibody. Total S‐EVs were determined as the number of detected particles bound to tetraspanin antibodies (CD9, CD63, CD81) and normalised to negative control IgG antibody. Frequency distribution of vesicle diameter and statistical analysis was performed using GraphPad Prism v9.0.1 and Excel. Significant differences between groups were identified with *P* ≤ 0.05.

### 2.5 Western blot

After conditioned media were collected, cells were detached from the flasks using Trypsin‐EDTA solution (Thermo Fisher) and washed with PBS prior to protein and RNA isolation. Cells were lysed using 10 mM Tris pH 7.8, 1 mM EDTA, 150 mM NaCl, 1% NP40, 1x Roche protein inhibitor cocktail on ice for 15 min and then spun for 15 min at 4°C at 10,000 *g*. The supernatant was collected and stored at ‐20 °C for further analysis.

EV samples (10 μg protein in PBS) or cellular proteins (30 μg protein) were analysed using Trans‐Blot Turbo Transfer System (Bio‐Rad). Membranes were probed with primary antibodies TSG101 (1:1000; BD Biosciences), Alix (1:1000, Cell Signalling), PSA (1:1000, Dako), EEA‐1 (1:1000, Abcam) followed with secondary antibodies HRP‐conjugated donkey anti‐rabbit IgG or HRP‐conjugated donkey anti‐mouse IgG (1:10,000; Millipore, Darmstadt, Germany) and visualised using the ChemiDoc MP System.

### 2.6 qRT PCR

Cellular RNA was isolated using RNeasy (Qiagen) according to the manufacturer's instructions. Primers were designed by Primer‐BLAST (NCBI) and ordered from Integrated DNA Technologies (USA) with sequences as follows: PSA (f)5′‐agtgcgagaagcattcccaac‐3′, (r)5′‐ccagcaagatcacgcttttgtt‐3′; and RPL32 (f)5′‐gcaccaccagtcagaccgatatg‐3′, (r)5′‐actgggcagcatgtgctttg‐3′. PSA expression was normalised to the housekeeping gene RPL32, and then expressed relative to the vehicle control at the same time point. Data were analysed with CFX ManagerTM (Bio‐Rad). Statistical analyses were performed using GraphPad Prism v9.0.1. Significant differences in gene expression between treated and untreated groups were identified with *P* ≤ 0.05.

### 2.7 Transmission electron microscopy (TEM)

Isolated S‐EVs were fixed with an equal volume of 3% glutaraldehyde in 0.1 M sodium cacodylate buffer for 15 min at room temperature, and loaded onto 200 mesh formvar coated copper grids. Samples were stained for 3 min with a 1% aqueous uranyl acetate and observed using a JEOL 1400 Transmission Electron Microscope, at an accelerating voltage of 100 kV. Images were acquired on a 2K F216 TVIPS camera.

### 2.8 Scanning electron microscopy (SEM)

Cells were cultured on round glass coverslips before fixation in 2.5% glutaraldehyde in 1 x PBS. Following fixation, samples were washed in 1 x PBS, dehydrated through a series of ethanol solutions (20%, 30%, 40%, 50%, 60%, 70%, 80%, 90%, 100%), and dried using hexamethyldisilazane (HMDS). Coverslips were mounted on aluminium stubs using adhesive carbon tabs and coated in platinum (Pt) using a Leica EM ACE 600 coater. Cells were subsequently imaged at 3 kV using a secondary electron detector on a Tescan MIRA3 field emission SEM. Images were prepared and arranged using Adobe Photoshop.

### 2.9 Flow cytometry

Flow cytometry was performed to indicate the surface expression of CD9 and CD63 on androgen manipulated AR expressing LNCaP prostate cancer cells. Cells were treated with androgens as described above and then harvested using 0.05% Trypsin‐EDTA, spun at 150 *g* for 5 min, resuspend with RPMI + 5% FBS and kept on ice. Cells were stained with Fluorescein‐conjugated anti‐human CD9 (clone #20930; R&D Systems) and anti‐human CD63 (clone AHN16.1/46‐4‐5; Ancell) according to the supplier's recommendations in fluorescence‐activated cell sorting (FACS) buffer (3% FBS in PBS) for 30 min at 4°C. Cells were then washed twice with FACS buffer and resuspended in FACS buffer containing propidium iodide (1.0 μg/ml; Sigma Aldrich). Isotype mouse IgG_2B_ antibody was used in isotype controls. Staining with propidium iodide was used to exclude dead cells during analysis and indicate the % viability. Singlet gate was used to select only single cells for further investigation of CD9 and CD63 expression. Fluorochrome‐conjugated antibody specific for human CD9 and CD63 was used to stain DHT‐ or ENZ‐ treated LNCaP cells, and median fluorescence intensity (MRI) of CD9 expression was determined. Live cells were gated based on propidium iodide exclusion and MRI of CD9 or CD63 expression was determined.

### 2.10 Isolation of EV‐derived small RNAs and identification

Isolation of extracellular and cellular miRNAs was performed using the miRVANA isolation kit (Thermo Fisher) according to the manufacturer's protocol. Detection of small RNAs and miRNAs was performed using the Agilent Bioanalyzer 2100 (Agilent Technologies) according to the manufacturer's protocol. Samples were analysed for total RNA using Agilent RNA 6000 Pico kit (Agilent Technologies) according to the manufacturer's protocol. Statistical analysis was performed using GraphPad Prism v9.0.1. Significant differences between the two groups were identified with *P* ≤ 0.05. Sequencing was performed through BGI (China) from a total of 1.5 ng (S‐EVs) or 14 ng (cells) total RNAs. An equal amount of isolated RNA from three biological repeats of S‐EVs and cell lysate was combined to generate small RNA libraries. Small RNA NGS libraries were prepared using the Illumina's TruSeq Small RNA Library Prep kit (Illumina) and single‐end sequenced at a read length of 50 nucleotides (nt). Fastq library files were processed using a custom‐designed pipeline. Adapters were removed using the Cutadapt tool (Marcel, 2011) at a maximum error rate of 0.1, a minimum overlap length of three. Reads that were not trimmed and reads shorter than 15 nt after trimming were removed from further analysis. To remove reads originating from ribosomal RNA, trimmed reads were mapped with high stringency against ribosomal sequences (Supplementary File 1) using Bowtie2 (Langmead & Salzberg, 2012) software with the following parameters: ‐N 0 ‐L 20 ‐i ‘S,1,0.50′ –n‐ceil ‘C,0′ –dpad 15 –gbar 4 –no‐1 mm‐upfront –end‐to‐end –score‐min ‘C,0′ ‐D 20 ‐R 3). High‐quality reads that did not map to rRNA sequences were mapped using the same settings against ncRNAdb.v10 (Hoogstrate et al., 2015), which contains curated sequences of human microRNA (miRNA), transfer RNA (tRNA), small nucleolar RNA (snoRNA), small nuclear RNA (snRNA), and small cytoplasmic RNA (cytRNA). SnoRNA and tRNA fragments (sdRNAs and tRFs) were identified using the FlaiMapper software (Hoogstrate et al., 2015) and manually curated as described (Olvedy et al., 2016). Reads that did not map to ncRNAdb entries were queried against a database of 32,000 known human piRNAs retrieved from RNA central (rnacentral.org). Remaining reads were mapped against the human genome hg38 using Bowtie2 using very‐sensitive pre‐set.

### 2.11 Data scaling and differential expression analysis

To compare expression levels, expression values were scaled at reads per million. Small RNAs with less than 10 mapping reads in any of the four libraries were excluded from further analysis. Small RNAs were considered differentially expressed if at least a 4‐fold difference was observed between the compared conditions.

### 2.12 IsoMir identification

Differentially expressed miRNAs identified by FlaiMapper were aligned to the precursor miRNA sequence (MiRBase release 22.1) (Kozomara et al., 2018). Entries with 3′‐end or 5′‐end additions or deletions were manually curated.

### 2.13 miRNA target prediction, validation and pathway analysis

Prediction of miRNA targets was performed using the microRNA Target Filter function in Ingenuity Pathways Analysis software (IPA, Ingenuity Systems, Redwood City, CA, USA), which utilises experimentally validated and predicted mRNA targets from TargetScan, TarBase, miRecords, and the Ingenuity Knowledge Base. Differentially expressed EV miRNAs were subjected to IPA Core Pathway analysis to identify cellular processes and functions that may be significantly altered.

Synthesis of cDNA was performed using 20 ng total RNA with miRCURY LNA Universal RT microRNA PCR, polyadenylation and cDNA synthesis kit (Qiagen, USA) according to the manufacturer's protocol, using Bio‐Rad T100 Thermal Cycler running for 60 min at 42°C and 5 min at 95°C. The commercially available miRCURY LNA miRNA PCR Assays were obtained from Qiagen (USA): hsa‐miR‐148b‐3p (5′UCAGUGCAUCACAGAACUUUGU), hsa‐miR‐19a‐3p (5′UGUGCAAAUCUAUGCAAAACUG), hsa‐miR‐361‐5p (5′UUAU‐CAGAAUCUCCAGGGGUAC), hsa‐miR‐99b‐5p (5′CACCCGUAGAACCGACCUUGCG), hsa‐miR‐181a‐5p (5′AACAUUCAACGCUGUCGGUGAGU). Samples isolated from three independent experiments were run in qPCR replicate. Real‐time qPCR was performed using a Bio‐Rad CFX384 Real‐Time Detection System (Applied Biosystems) at the following conditions: 95°C for 2 min, thereafter 40 amplification cycles at 95°C for 10 s, 56°C for 1 min (ramp‐rate 1.6°C/s). Expression was normalised to miR‐181a that was found equally in all samples and then expressed relative to the vehicle control at the same time point. Data were analysed with CFX Manager™ (Bio‐Rad). Statistical analysis was performed using GraphPad Prism v9.0.1. Significant differences in gene expression between two groups were identified with *P* ≤ 0.05.

### 2.14 Expression analysis from tissue‐derived samples

Expression levels of miRNA upregulated upon DHT stimulation in cell cultures were examined in tissue‐derived samples from the archive Erasmus MC, Rotterdam prostate cancer patient cohort with over 15 years of clinical follow‐up, approved by the Erasmus MC Medical Ethics Committee according to the Medical Research Involving Human Subjects Act (MEC‐2004‐261) (Martens‐Uzunova et al., 2012). Briefly, prostate cancer (PCa) and normal adjacent tissue (NAP) were obtained during radical prostatectomy. Transurethral resection of the prostate (TURP)‐PCa and TURP‐BPH samples were obtained from material collected during TURP procedure. MiRNA expression was acquired using Human miRNA V2 microarrays (Agilent Technologies) that contain probe sets for 723 human miRNAs from the Sanger MiRBase, v.10.1. Clinical parameters, patient characteristics and experimental procedures have been described previously (Martens‐Uzunova et al., 2012) and a summary is provided in Supplementary file 4. Differential expression of miR‐19a‐3p and miR‐361‐5p was evaluated using GraphPad Prism v9.0.1.

## 3 RESULTS

### 3.1 The heterogeneity of circulating S‐EVs in prostate cancer

**FIGURE 1:**
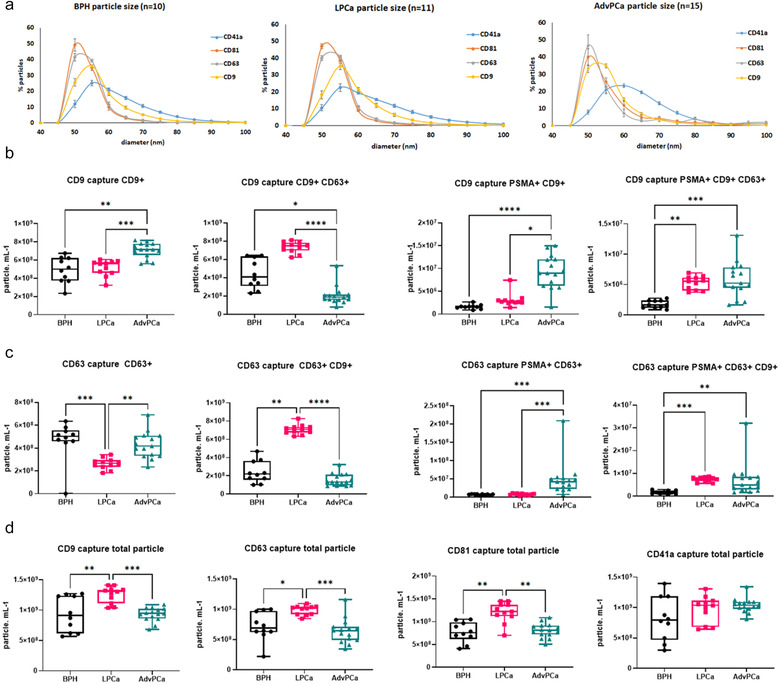
Circulating S‐EV profile in plasma from benign (BPH), localised (LPCa) and advanced prostate cancer (AdvPCa) patients. (a) Size measurement of S‐EVs from BPH, LPCa and AdvPCa captured using CD63, CD9, CD81 and platelet EV marker CD41a. Data are mean ± SEM. Vesicle count using CD9 (b) and CD63 (c) captured antibody on plasma‐derived S‐EVs and detected using CD9, CD63 or PSMA antibodies. (d) Total amount of S‐EVs captured using tetraspanin antibodies. PSMA: Prostate Specific Membrane Antigen. Data distribution is represented by box plots, where whiskers represent the minimum and maximum values, and the box extends from the 25^th^ to the 75^th^ percentile with the 50^th^ percentile (median) value shown. P‐values are indicated by asterisk (Kruskal‐Wallis test, *P < 0.05, **P < 0.01, ***P < 0.001, **** *P* < 0.0001).

To investigate whether S‐EVs are indeed heterogeneous in prostate cancer, we first identified the characteristics of S‐EVs based on their size, total number and expression of tetraspanins found in circulating S‐EVs. The S‐EV profiles from advanced prostate cancer (AdvPCa, *n* = 15) were compared with BPH patients (*n* = 10) and localised PCa (LPCa, *n* = 11). S‐EVs were captured using tetraspanin specific antibodies. Analysis using ExoView shows the presence of CD63, CD9 and CD81 on captured vesicles. In AdvPCa, the CD63+ and CD81 positive (CD81+) S‐EVs were smaller with a diameter of 50 nm (46.6% for CD63 and 39.8% CD81), while CD9+ S‐EVs were found to be both 50 nm (33.1%) and 55 nm (34.8%) (Figure 1a). The CD41a platelet‐derived S‐EVs are larger vesicles with 60 nm in diameter (23.3%). S‐EVs from BPH patients showed a similar profile; however, CD9+ and CD41a positive (CD41a+) S‐EVs are mostly vesicles with 55 nm in diameter (35.9% and 25.1%, respectively). In LPCa, both 50 nm and 55 nm of CD63+ S‐EVs were observed (40 and 40.7%, respectively, and a single peak of CD9+ S‐EVs with 55 nm in diameter (35%). In all BPH, LPCa and AdvPCa samples, we consistently detect a higher percentage of 55 nm S‐EVs in CD9+ S‐EVs (Figure 1a).

We then assessed the heterogeneity of S‐EVs by detection of S‐EVs using fluorescence‐ tagged tetraspanin antibodies. The CD9+ S‐EVs were more abundant in AdvPCa samples (4.91E+08 particle/ml, Figure 1b) and significantly higher than CD9+ S‐EVs from BPH patients (**P < 0.01) and LPCa (***P < 0.001). Additional labelling using prostate cancer‐specific PSMA antibodies increased this difference. PSMA positive (PSMA+) S‐EVs that are also CD9+ in AdvPCa patients were increased by 5.74‐fold compared with BPH and 2.99‐fold compared with LPCa. PSMA+ CD9+ CD63+ S‐EVs measurement also showed increased by 3.34‐fold in AdvPCa patients versus BPH patients (Figure 1b, **P < 0.01). S‐EVs captured using CD63 antibodies show a significant difference between AdvPCa versus LPCa patients in those that are also CD9+ (CD9+ CD63+; Figure 1c, ****P < 0.0001), but not significant between AdvPCa and BPH. Detection using PSMA antibody also increased cancer detection specificity as the CD63 captured S‐EVs from LPCa and AdvPCa were significantly higher than in benign samples (PSMA+ CD63+ CD9+; Figure 1c, **P < 0.01, ***P < 0.001). CD9+ CD63+ S‐EVs was higher on both S‐EVs captured using CD9 antibody detected with CD63 fluorescence tagged antibody and those that were captured using CD63 antibody and followed by detection using CD9 fluorescence‐tagged antibody (Figure 1b and 1c). Using either CD9, CD63 or CD81 antibodies to capture S‐EVs, LPCa has more abundance circulating S‐EVs in comparison with either BPH or AdvPCa samples (Figure 1d, *P < 0.05, **P < 0.01, ***P < 0.001). While the cause of this difference is not clear, these data support the robustness of detection for prostate cancer‐specific circulating S‐EVs based on assessment of the heterogeneity at the single EV level.

### 3.2 The effect of androgens on secreted S‐EVs

**FIGURE 2:**
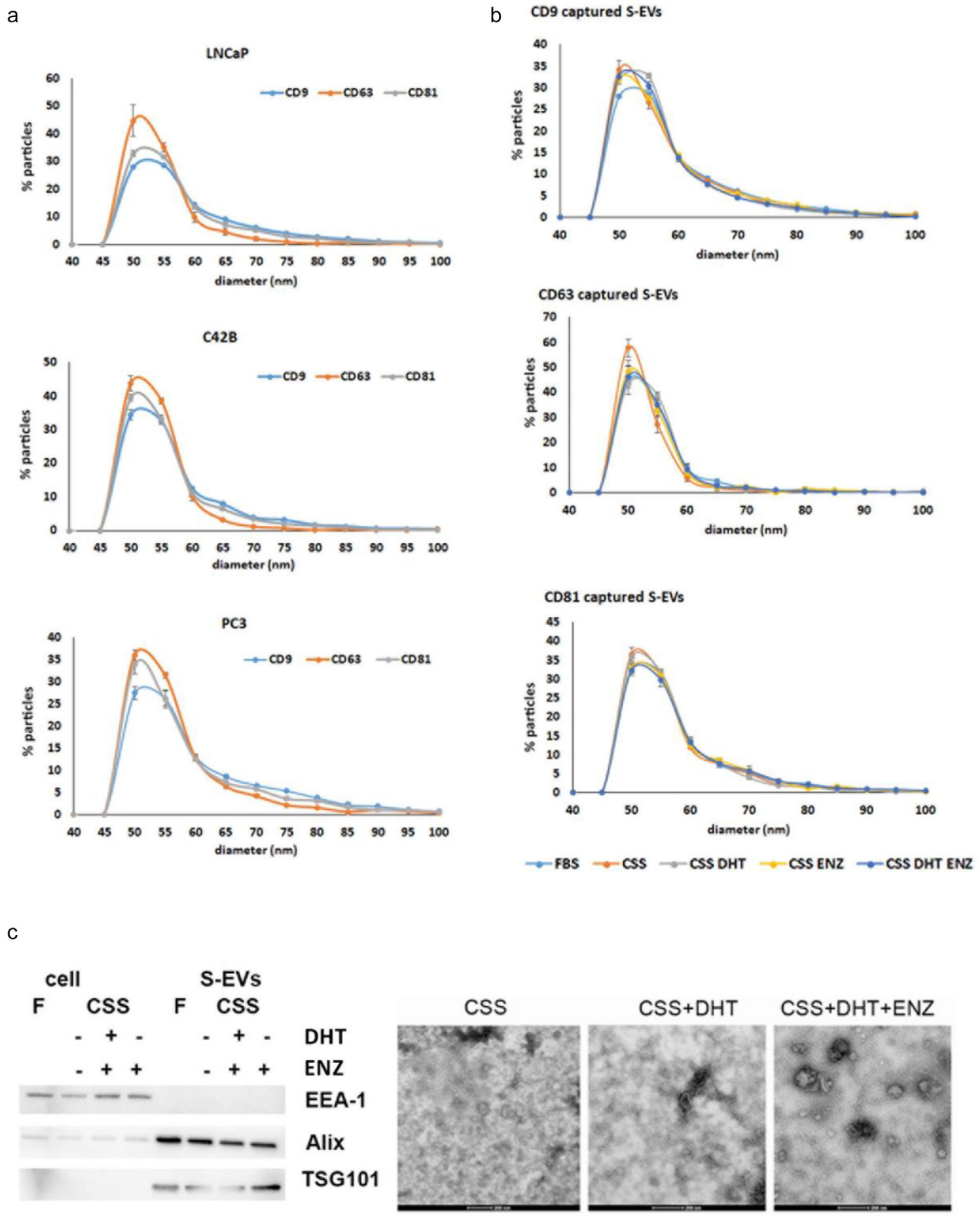
S‐EV characterisation from prostate cancer cells. (a) Frequency distribution of the size of S‐EVs from AR positive LNCaP and C42B cells, and AR negative PC3 cells; (b) Treatment with androgens alters the diameter of S‐EVs secreted by LNCaP cells; (c) A representative western blot of cells and S‐EVs probed with positive markers TSG101 and Alix, or a negative marker EEA‐1. TEM images of S‐EVs isolated from LNCaP cells, treated with androgen DHT, or in the presence of androgen antagonist ENZ. Scale bar: 200 nm. Data of frequency distribution are mean ± SEM (*n* = 3 independent experiments). F: FBS, CSS: charcoal‐stripped serum, DHT: dihydrotestosterone, ENZ: enzalutamide.

We previously showed that DHT affects S‐EV secretion from AR positive LNCaP and DUCaP cells (Soekmadji et al., 2016). DHT does not induce the secretion of the prostate cancer serum marker, Prostate Specific Antigen (PSA) and AR protein in S‐EVs (Soekmadji et al., 2016). Other EV markers such as TSG101 and Alix are also detected in S‐EVs in equal abundance irrespective of DHT or ENZ treatments (Soekmadji et al., 2016). We analysed the heterogeneity profile of S‐EVs from AR expressing cells, LNCaP and C42B, as well as AR negative PC3 cells. Captured using tetraspanin antibodies indicate that CD63+ S‐EVs are mostly around 50 nm in diameter, while CD9+ and CD81+ S‐EVs are 50 and 55 nm in diameter in LNCaP and C42B S‐EVs (Figure 2a top and middle panels). AR negative PC3 also secreted CD9+, CD81+ and CD63+ S‐EVs, with the highest frequency of PC3‐derived CD81+ S‐EVs are 50 nm in diameter (Figure 2a, lower panel).

It has been reported that culture conditions could affect the secretion profile of S‐EVs. There was no significant effect of our different experimental treatments on cell viability (%) of LNCaP cells grown under different conditions, as cell viability in the presence of either FBS, CSS, CSS + DHT, CSS + ENZ, and CSS + DHT + ENZ are similar(80.80±6.123, 79.20±7.181, 83.28±5.331, 78.63±7.754, and 83.05±4.448, respectively (mean ± SEM)). We dissected the effect of androgens by firstly characterising the size of vesicles from LNCaP and C42B. Using CD9 captured antibody, we observed similar frequency for 50 and 55 nm S‐EVs in DHT treated LNCaP cells (31.7% and 32.8%, respectively), while those that were grown without DHT (in CSS), showed a single peak at 50 nm (34.2 %, Figure 2b). These changes were not observed on CD63 and CD81 S‐EVs. Further, ENZ treatment does not completely reduce the abundance of 55 nm S‐EVs, with similar frequency was found on 50 and 55 nm S‐EVs (32% and 30.5%, respectively). A similar trend was also observed in C42B cells (Figure S1A). DHT indeed induced the formation of larger S‐EVs as observed by TEM imaging (Figure 2c, right panel; western blot of isolated S‐EVs are shown on the left panel). Further, exposure to ENZ did not eliminate the presence of these vesicles.

**FIGURE 3:**
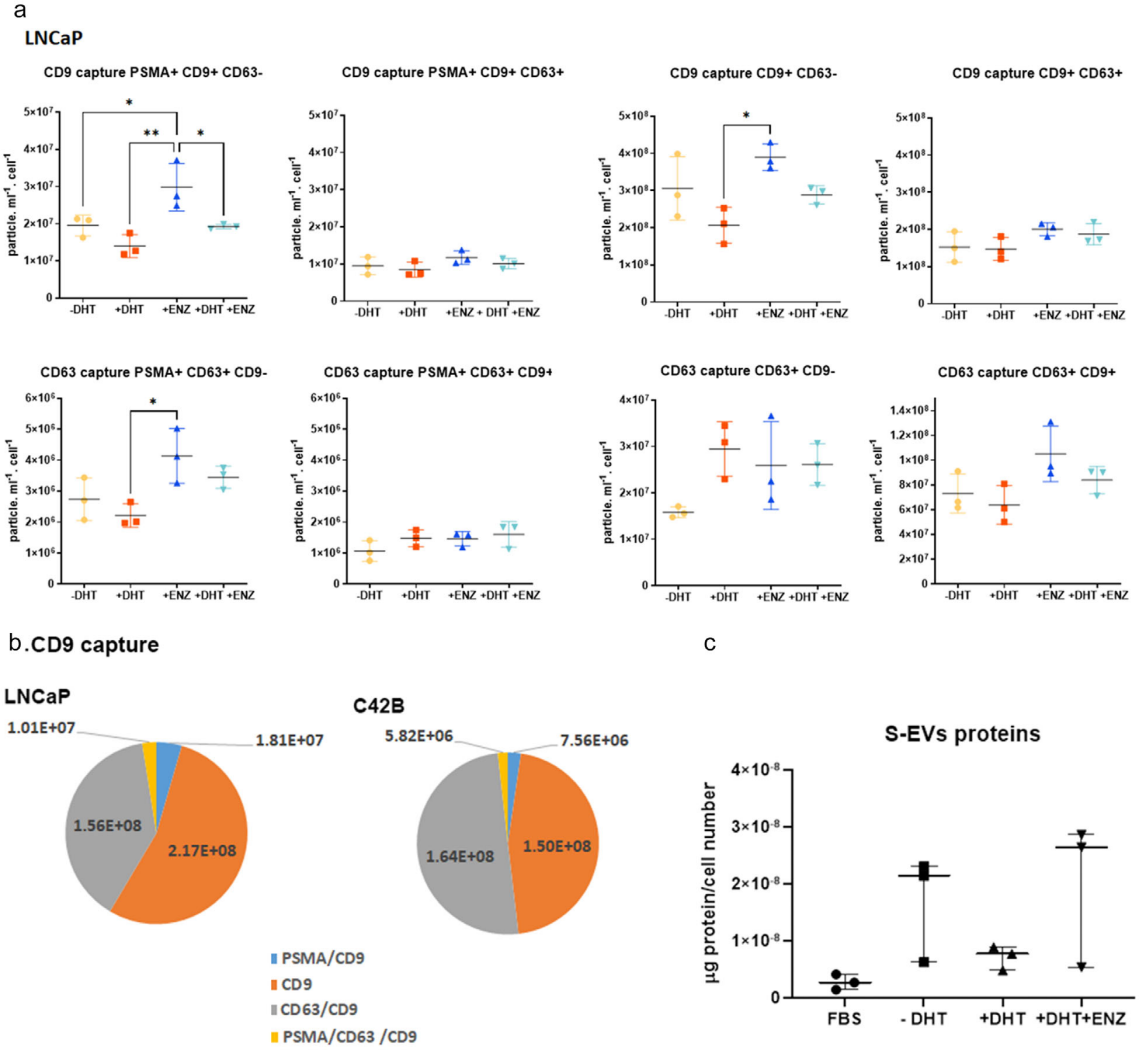
Heterogeneity of S‐EVs from prostate cancer cells under androgen manipulation. (a) S‐EVs were captured using CD9 or CD63 specific antibodies, and labelled by fluorescence tagged CD9, CD63 or PSMA antibodies. LNCaP conditioned medium was collected from three independent experiments. (b) CD9+ S‐EVs profile for LNCaP (top) and C42B cells (lower) grown in FBS. Data are mean from three independent experiments. (c) The abundance of S‐EV protein cargo upon androgen manipulation normalised by LNCaP cell number at the end of experiments. P‐values are indicated by asterisk (ordinary one‐way ANOVA, multiple comparison, *P < 0.05, **P < 0.01).

We sought to understand the role of androgens on the heterogeneity of S‐EVs. While similar trends were observed on S‐EVs captured using CD9 or CD63 antibodies, DHT increased the CD63+ S‐EVs population compared to cells grown in CSS only (1.86‐fold; Figure 3a CD63 capture CD63+ CD9‐), but not on S‐EVs that are CD9+ CD63+; or CD9+ CD63+ PSMA+. Simultaneously, capturing using CD9 or CD63 antibodies showed that PSMA+ CD9+ or PSMA+ CD63+ S‐EVs were significantly reduced in the presence of androgen DHT compared to the inhibitor ENZ (Figure 3a, *P < 0.05, **P < 0.01). The CD9+ S‐EVs profile of another AR positive cells, C42B, was similar to LNCaP (Figure 3b, see also the androgen effect on C42B S‐EVs in Supplementary Figure 1B). Total protein content of total S‐EVs across the treatment groups did not show significant difference (Figure 3c), implicating that assessment on S‐EV heterogeneity is more informative to indicate response to androgens.

**FIGURE 4:**
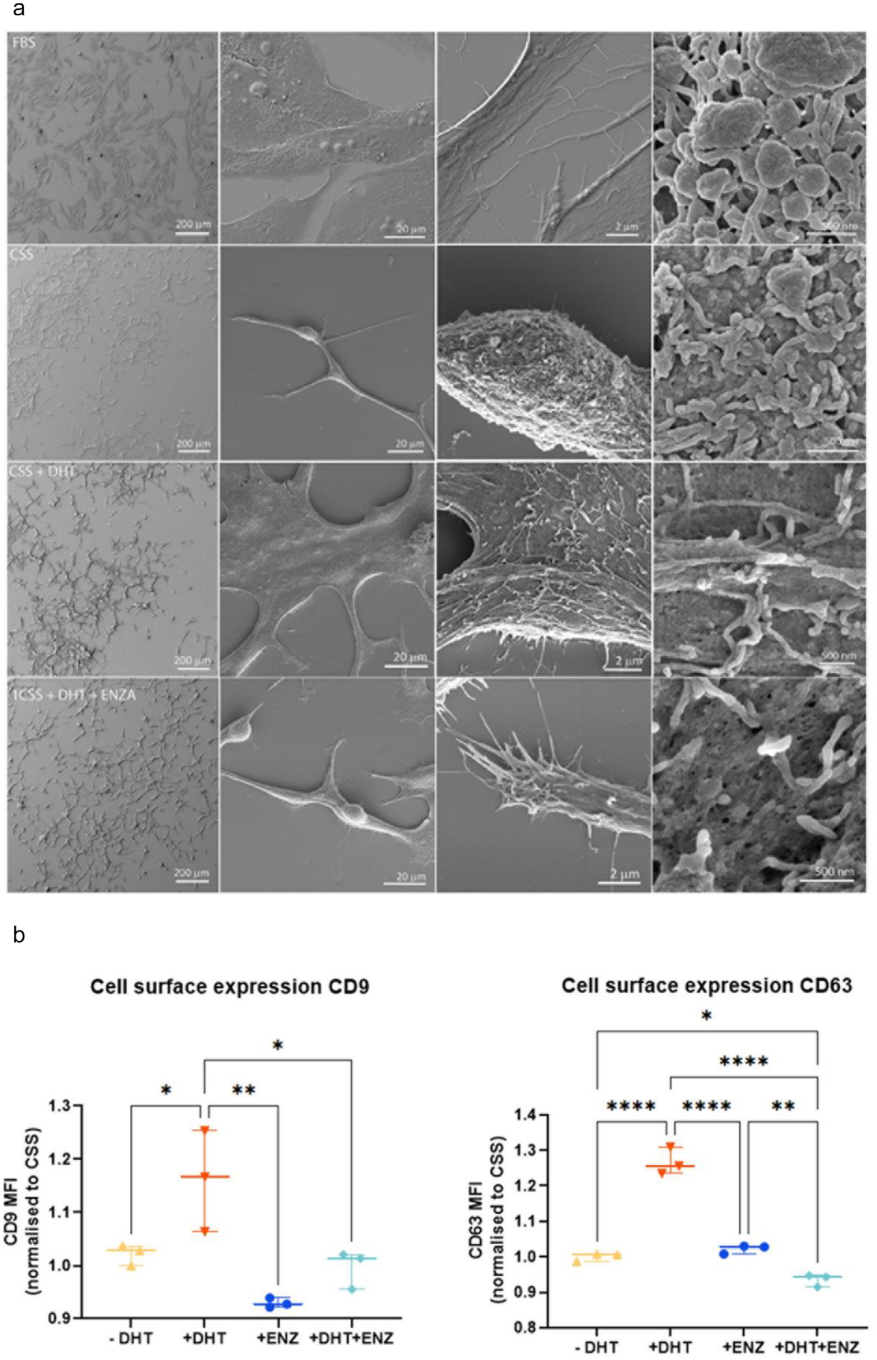
(a). Scanning Electron Microscope images on LNCaP cells under treatment with androgens. LNCaP cells are treated with androgen DHT, or in the presence of androgen antagonist ENZ. Scale bar: 200 μm, 20 μm, 2 μm and 500 nm (left to right panel). CSS: charcoal‐stripped serum, DHT: dihydrotestosterone, ENZ: enzalutamide; (b) Cell surface marker of CD9 (median fluorescence intensity; MFI, left panel) and CD63 (right panel) on LNCaP cells treated with FBS, CSS, CSS + DHT or ENZ, and CSS+ DHT+ ENZ. P‐values are indicated by asterisk (ordinary one‐way ANOVA, multiple comparison, *n* = 3 independent experiments; *P < 0.05, **P < 0.01,****P < 0.0001).

Imaging analysis such as Atomic Force Microscopy and SEM on the cell surface of parental cells has been utilised to help characterise secretion of EVs (Silva et al., 2018; Taylor et al., 2020). Analysis of the cell surface of LNCaP cells using SEM imaging reveals that the absence of DHT in medium supplemented with CSS activates the budding of EVs from the cell surface (Figure 4a). On the other hand, the addition of DHT allows cells to increase their surface area and induces the presence of plasma membrane protrusion filopodia as also observed for cells grown in medium supplemented with full serum, and for any DHT treated cells (with or without ENZ). We performed quantitative measurement on the presence of tetraspanin EV markers on the plasma membrane using flow cytometry. We found a significant increase in CD9 and CD63 mean fluorescence intensity (MFI) on the surface of LNCaP cells in cells treated with DHT in comparison to cells grown in CSS, CSS+ ENZ or CSS + DHT + ENZ (Figure 4b; *P < 0.05, ** *P* < 0.01; ****P < 0.0001, *n* = 3 biological replicates).

### 3.3 Global changes in small RNA signatures

**FIGURE 5:**
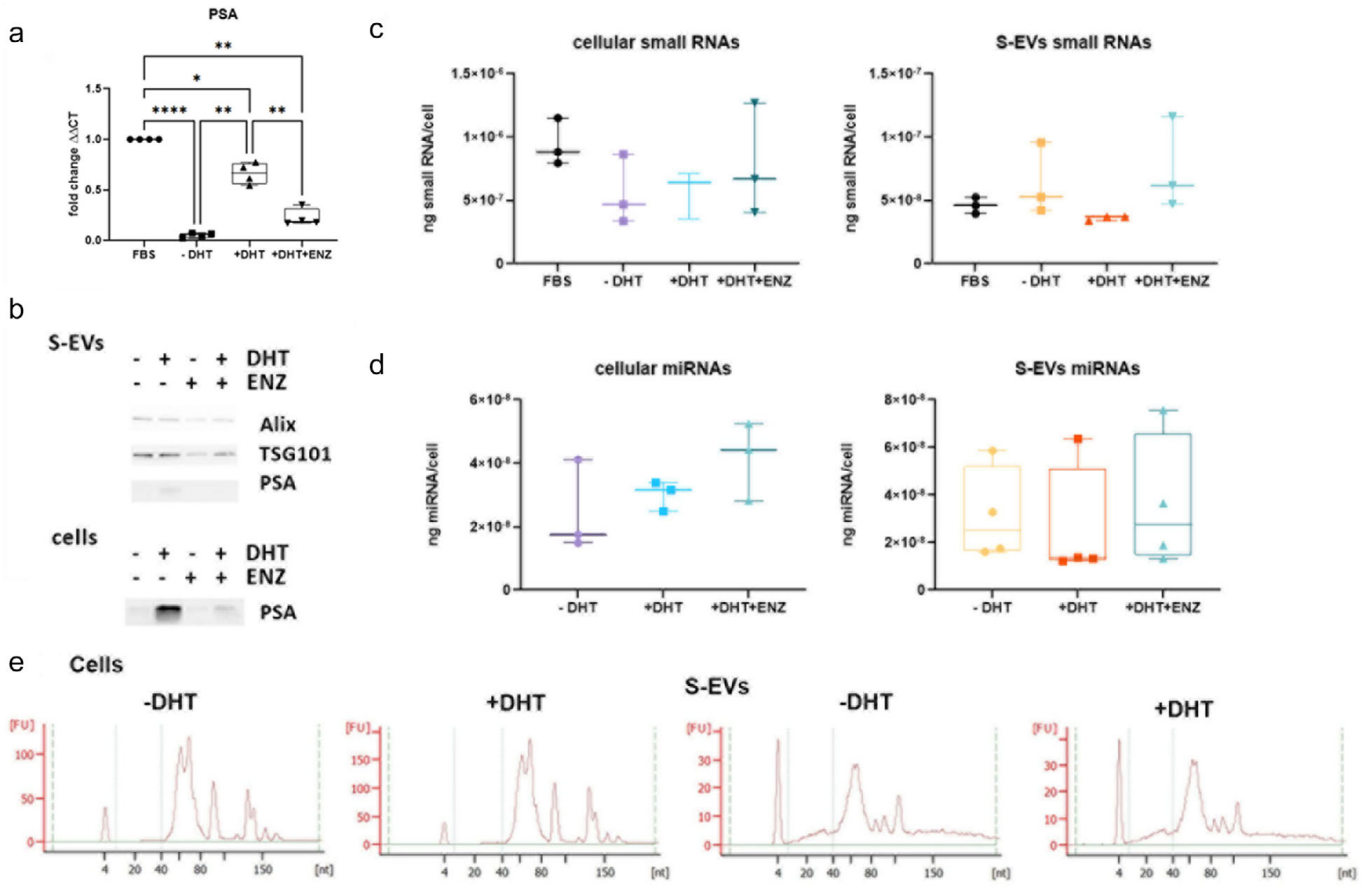
The overall profile of small RNA derived from S‐EVs or parental LNCaP cells. (a) Quantitative RT PCR analysis of cellular mRNA transcript of PSA to indicate activation of AR signalling axis (RM‐one way ANOVA, *n* = 4 independent experiments, *P < 0.05, **P < 0.01,****P < 0.0001); (b) A representative western blot of S‐EVs; (c) and (d) Yield of overall small RNAs (c) and miRs (d) from parental cells and isolated S‐EVs (*n* = 3–4 independent experiments). (e) Representative small RNA profile from parental cells and isolated S‐EVs with and without DHT treatment, analysed using Agilent Bioanalyzer.

We isolated RNA from cells and matching S‐EVs to evaluate the effect of androgens on small ncRNA in terms of cellular expression and EV cargo content. As a control experiments, treatment with DHT significantly increased the PSA mRNA and protein expression in LNCaP cells (Figures 5a, *P < 0.05, ** *P* < 0.01; ****P < 0.0001, *n* = 4 biological replicates; and Figure 5b, ponceau stained blot in Figure S2). Next, we isolated the small RNA fraction from cells and S‐EVs to investigate the effect of androgens on the overall small RNA profiles. We found that treatment with DHT or ENZ did not significantly alter the amount and comprehensive profile of small RNAs and miRNA isolated from parental cells or in S‐EVs (Figure 5c–e).

**FIGURE 6:**
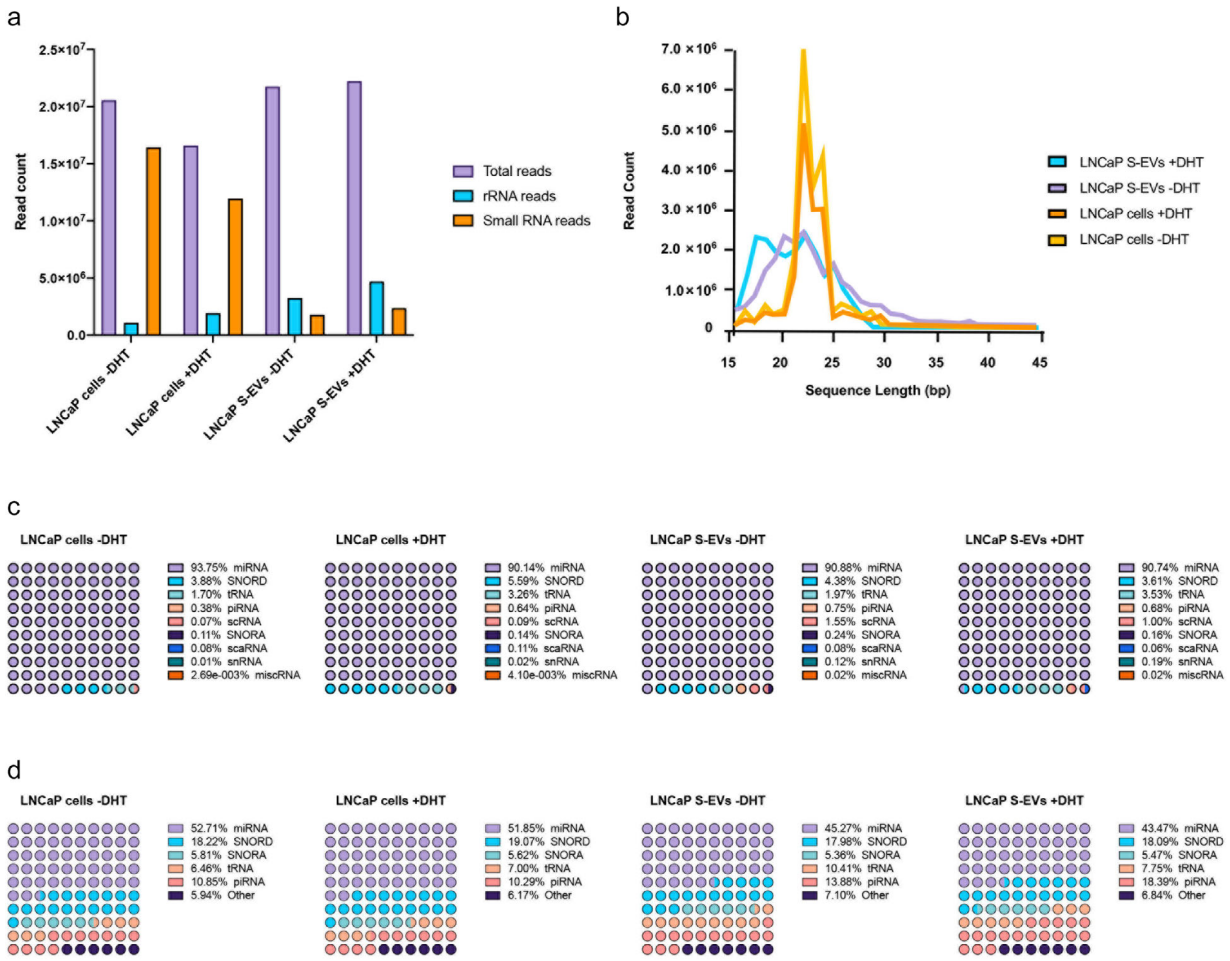
Overall characteristics and content of the sequencing libraries. (a) Number of total reads, and reads mapped to rRNA and small RNA; (b) Sequence read length distribution per generated library; (c) Relative abundance of read counts per small RNA species; (d) Relative abundance of small RNA species.

To investigate how androgen stimulation affects the small RNA content of S‐EVs produced by prostate cancer cells, we used next‐generation sequencing to determine the small RNA transcriptome of LNCaP cells and S‐EVs upon hormone stimulation. We generated four small RNA sequencing libraries, two from LNCaP cells grown in CSS with or without DHT, and two from EVs isolated from the corresponding culture media. After removing reads originating from ribosomal RNA, we mapped the sequencing reads against a database of known small RNAs, that was previously described (Hoogstrate et al., 2015). As expected, libraries constructed from cellular RNA contained up to 80% of filtered reads mapping to small RNA (Figure 6a) and showed a narrow size distribution between 20 and 25 nt (Figure 6b). In libraries constructed from S‐EVs extracted RNA (evRNA), small RNA mapping reads represented up to 10% of all filtered reads (Figure 6a) with a broader size distribution between 16 and 30 nt (Figure 6b). Interestingly, the overall GC content of the evRNA libraries (53%) was higher than the GC content of libraries constructed from cellular RNA (45–46%).

The majority of reads mapped to microRNAs (90–93%), followed by C/D‐box snoRNAs (SNORD, 3–6%), tRNAs (2–4%) and PiWi interacting RNAs (piRNAs, 0.4–0.8%) (Figure 6c). The relative proportion of different detected small RNA species was relatively uniform across all four libraries (Figure 6d). About half of the detected small RNAs were microRNAs (43–53%) followed by SNORDs (18–19%), piRNA (10–14%), tRNA fragments (tRFs, 6–11%) and H/ACA‐box snoRNAs (SNORAs, ‐6%), The remaining detected small RNAs included small Cajal body RNAs (scaRNA), as well as small nuclear RNAs (snRNA), and small cytoplasmic RNAs (scRNAs) including vault RNAs, fragments from the RNA components of ribonuclease P, telomerase, signal recognition particle, Ro60 particle, 7SK, and Small NF90 (ILF3) associated RNAs (SNAR). We were able to detect more different piRNAs in S‐EVs compared to cells. In contrast, more miRNA species were detected in cells (Figure 6d, Table 1 and Table 2).

**TABLE 1:** Detected small RNAs per small RNA type

	LNCaP cells		LNCaP S‐EVs	
	−DHT	+DHT	−DHT	+DHT
miRNA	408	378	287	286
SNORD	141	139	114	119
SNORA	45	41	34	36
tRNA	50	51	66	51
piRNA	84	75	88	121
scaRNA	16	13	9	10
scRNA	9	10	10	11
miscRNA	2	3	5	4
ribonuclease P RNA	1	1	1	1
TERC	1	1	1	1
RN7SL	2	2	2	2
RNY	3	3	3	3
VTRNA	2	3	3	4
SNAR	3	3	5	3
RNU	15	15	15	16
RN7SK	1	1	1	1

**TABLE 2:** Read count distribution among different small RNA types

	LNCaP cells		LNCaP S‐EVs	
	−DHT	+DHT	−DHT	+DHT
miRNA	15,323,830	10,661,179	1,620,944	2,145,995
SNORD	634,634	661,063	78,149	85,308
tRNA	278,497	385,205	35,204	83,442
piRNA	62,383	76,121	13,459	16,169
scRNA	12,229	10,606	27,556	23,707
SNORA	17,938	16,190	4,300	3,801
scaRNA	12,905	13,386	1,350	1,502
snRNA	2389	2701	2227	4582
ribonuclease P RNA	277	306	149	337
TERC	162	179	175	57
Other small RNA	439	485	324	394

### 3.4 Changes in small evRNA abundance

**FIGURE 7:**
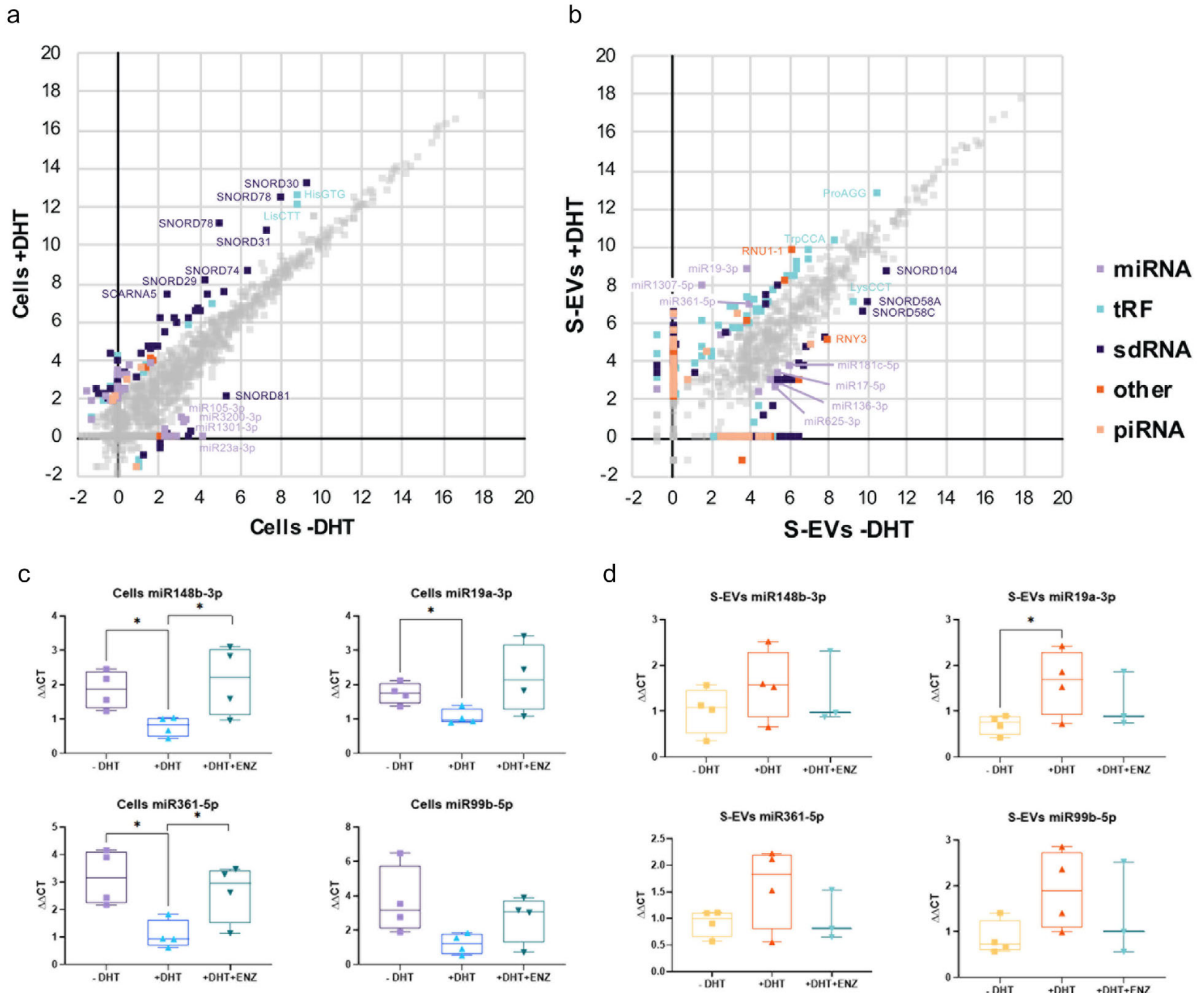
Differential expression of small RNA upon androgen stimulation. (a) and (b) Graph of LNCaP cells (a) and S‐EVs isolated from conditioned medium (b). Axes are in log2 scale. Differentially expressed small RNAs (expression fold change > 4), are coloured according to the legend. Small RNAs with a change in expression level less than 4‐fold are in grey; c) and d) Validation of cellular (c) and S‐EVs (d) miRNAs under androgen manipulation. QRT PCR analysis on paired parental and isolated S‐EVs from LNCaP cells treated with DHT +/‐ androgen antagonist ENZ. P‐values are indicated by asterisk (unpaired t‐test, *n* = 3–4 independent experiments; *P < 0.05).

To identify small evRNAs responsive to androgen stimulation, we applied reads per million (RPM) scaling and compared the RPM counts of individual small evRNAs. To reduce noise, only small RNAs that have been identified by at least 10 sequencing reads in any one of the tested conditions were included in the analysis. Small RNAs with a linear fold change of four or higher were considered as androgen‐responsive. After filtering, 143 small RNAs remained and were assigned as androgen‐responsive in LNCaP cells. While androgen addition had only a minor impact on the cellular expression levels of microRNAs, we observed substantial increase in the levels of several sdRNAs and tRFs (Figure 7a, Table 3, and Supplementary file 1).

**TABLE 3:** Differentially expressed small RNA

	LNCaP cells			LNCaP S‐EVs		
sncRNA Type	FC > = 4	Up	Down	FC > = 4	up	down
miRNA	37	26	11	169	99	70
piRNA	6	5	1	77	50	27
tRFs	32	25	7	113	92	21
sdRNA	58	47	11	140	76	64
SNORD	44	36	8	91	50	41
SNORA	8	5	3	38	20	18
scaRNA	6	6	0	11	6	5
Other	10	6	4	44	27	17
snRNA	8	6	2	25	13	12
scRNA	2	0	2	19	14	5
Total	143	109	34	543	344	199

Interestingly, a higher number of androgen‐responsive small RNAs in LNCaP S‐EVs were detected, with 543 small RNAs regulated by androgen addition (Figure 7b, Table 4). These included 169 miRNAs, 140 sdRNAs (91 SNORD‐derived, 38 SNORA‐derived, and 11 scaRNA‐derived), 113 tRFs, 77 piRNAs, as well as 19 scRNAs and 25 snRNA. Small evRNAs with most positive response upon androgen stimulation were miR‐1307‐5p (84‐fold), miR‐19a‐3p (31‐fold), miR‐361‐5p (8‐fold), piR‐50603 (9‐fold) (as well as fragments derived from SNORD14E (6‐fold), SNORD68 (7‐fold), SNORD116 (5‐fold), the spliceosomal RNA U1 (RNU1‐1, 13‐fold), and 3′‐tRFs originating from the mature tRNAs ProAGG (5‐fold) and TrpCCA (4‐fold) (Figure 7b). Small evRNAs with pronounced negative response to androgens included miR‐17‐5p (‐4‐fold), miR‐136‐3p (‐6‐fold), miR‐181c‐5p (‐5‐fold), and miR‐ 625‐3p (‐4‐fold), piR‐31355 (‐5‐fold) the 5′‐tRF LysCTT (‐4‐fold), as well as sdRNA originating from SNORD58A (‐7‐fold), SNORD58C (‐9‐fold) and SNORD104 (‐5‐fold).

**TABLE 4:** Differentially expressed canonical miRNAs and isoMiR forms

	Detected in S‐EVs number *(%)*	Detected in cells number *(%)*
Canonical miRs	52 *(35 %)*	10 *(27 %)*
IsomiRs	81 *(55 %)*	15 *(41 %)*
Shorter 3′‐end	33 (22 %)	12 *(32 %)*
Shorter 5′‐end	4 *(3 %)*	0 *(0 %)*
Longer 3′‐end	23 *(16 %)*	1 *(3 %)*
Longer 5′‐end	7 *(5 %)*	1 *(3 %)*
Shifted 5′‐end → 3′‐end	8 *(5 %)*	0 *(0 %)*
Shifted 5′‐end ← 3′‐end	4 *(3 %)*	1 *(3 %)*
Shorter on both ends	2 *(1 %)*	0 *(0 %)*
Opposite strand	6 *(4 %)*	2 *(5 %)*
Trailers	6 *(4 %)*	5 *(14 %)*
Stem Loops	3 *(2 %)*	5 *(14 %)*
Total	148 *(100 %)*	37 *(100 %)*

### 3.5 IsoMiR identification

Most miRNA species can produce mature functional miRNA products from either arm of the hairpin precursor. In addition, the 5′‐ and 3′‐ends of mature miRs can differ from the canonical miR sequence annotated in public repository databases, giving rise to shorter or longer isomiRs. The variability of the 5‐end is profoundly lower as it reflects the functional importance of the miR seed sequence. The 3′‐end of the mature miR is usually more prone to variation. Accordingly, the sequences of identified miRNA species were compared against their canonical counterparts as annotated in MirBase (V22.1: October 2018) in order to evaluate the abundance of miRNA isoforms in LNCaP S‐EVs. We restricted isomiR identification only to miRNAs that were differentially expressed upon androgen stimulation.

Interestingly, isomiRs were twice as abundant as the canonical miRNAs in both cells and S‐EVs (Table 4). From the 185 miRNAs differentially expressed either in EVs or cells, only 62 had a sequence identical to the canonical miRNA form, while 96 were identified as shorter or longer isoforms. As expected, one nucleotide (nt) length variation in the 3′‐end was the most commonly detected isoform type (Supplementary file 2).

In addition to isomiRs, we found 8 miRNAs that were unannotated in MirBase, which originate from known miRNA precursors but from the arm opposite to the canonical mature miRNA (Table 5). The FlaiMapper algorithm identifies the predominant form and ends of small RNAs based on the abundance of reads mapping to the corresponding precursor and the 5′‐ and 3′‐consensus position of sequence read termini. Therefore, we propose that in our dataset, these miRNAs represent the most abundant mature miRNA forms. Finally, 34 miRNA sequences mapped to either the stem‐loop region (Carrington, 2003) or trailer sequences produced during the miRNA precursor stem‐loop processing.

**TABLE 5:** MicroRNAs originating from the opposite arm of known miRNA precursors

ID	Detected miRNA sequence	Length (nt)	Detected in	Annotation
id 21	AGCTTCTTTACAGTGTTGCCT	21	S‐EVs	hsa‐miR‐107‐5p
id 69	CAGAGTCTCGTTCTGTTGCCC	21	S‐EVs	hsa‐miR‐1273c‐3p
id 335	ACTTCCCCCACCTCACTGCCC	21	S‐EVs	hsa‐miR‐3138‐5p
id 344	CCTGTAATCCCAGCATTT	18	S‐EVs	hsa‐miR‐3159‐3p
id 379	CCTCATCTGTCTGTTGGGCT	20	cells	hsa‐miR‐326‐5p
id 535	AAACTCAGTAATGGTAACGGTTT	23	cells	hsa‐miR‐451b‐3p
id 713	CCCCTCAGTCCACCAGAGCCCGGA	24	S‐EVs	hsa‐miR‐760‐5p
id 716	CGGGTCTCGGCCCGTACAGTCCGG	24	S‐EVs	hsa‐miR‐762‐5p

### 3.6 miRNA target gene analysis

An IPA Core Analysis demonstrated that androgen‐responsive EV miRNAs are significantly enriched in networks associated with reproductive system disease and cancer, and participate in molecular and cellular functions associated with cell cycle, development, proliferation and morphology (Table 6). To identify possible target genes that may be affected by androgen‐responsive miRNAs, we performed IPA miRNA Target Filter Analysis for miR‐19a‐3p, miR‐361‐5p miR‐1307‐5p that were the highest upregulated miRNAs upon androgen stimulation. When the highest prediction confidence level was applied, and only experimentally validated targets were permitted, the analysis algorithm returned 12 targets for miR‐19a‐3p (ALOX5, BCL2L11, BMPR2, CCN2, CCND1, ERBB4, ESR1, HIPK3, MYLIP, NR4A2, PTEN, and THBS1) and 1 for miR‐361‐5p (AICDA). No experimentally observed targets were identified for miR‐1307‐5p. Therefore, we investigated the interaction networks of each one of the three miRNAs and looked for genes that are targeted by more than one miRNA (Supplementary file 3). Only one gene, MDM4, was a common target for all three miRNAs. MiR‐19a‐3p and miR‐361‐5p shared additional 44 common targets, while only limited overlap was found between the targets of miR1307‐5p and miR‐19a‐3p (ITSN1 and RPS6KA2) and miR‐1307‐5p and miR‐361‐5p (RNF4) in addition to MDM4. Validation of identified miRNAs was performed by qRT PCR using a Locked Nucleic Acid (LNA) system. The results showed that DHT increased the presence of miR‐19a‐3p in S‐EVs, while DHT downregulated its expression in cells (Figure 7c and d). The effect of ENZ on suppressing the cellular expression of miR‐361‐5p was evident; however, while miR‐361‐5p expression in S‐EVs was increased (similar to that seen for miR‐19a‐3p), this did not reach statistical significance.

**TABLE 6:** Disease association and molecular and cellular functions significantly enriched with androgen‐responsive S‐EV derived miRNAs

Name	*P*‐value range	Number of molecules
Diseases and disorders		
Organismal injury and abnormalities	4.96E‐02 ‐ 2.07E‐36	56
Reproductive system disease	8.91E‐03 ‐ 2.07E‐36	42
Cancer	4.96E‐02 ‐ 9.54E‐20	38
Connective tissue disorders	4.68E‐02 ‐ 6.16E‐12	15
Inflammatory disease	4.68E‐02 ‐ 4.07E‐11	25
Molecular and cellular functions		
Cell cycle	4.36E‐02 ‐ 4.67E‐07	5
Cellular development	4.40E‐02 ‐ 1.84E‐05	30
Cellular growth and proliferation	4.40E‐02 ‐ 1.94E‐05	26
Cell morphology	4.89E‐02 ‐ 2.28E‐05	4

### 3.7 Expression analysis of tissue‐derived samples

**FIGURE 8:**
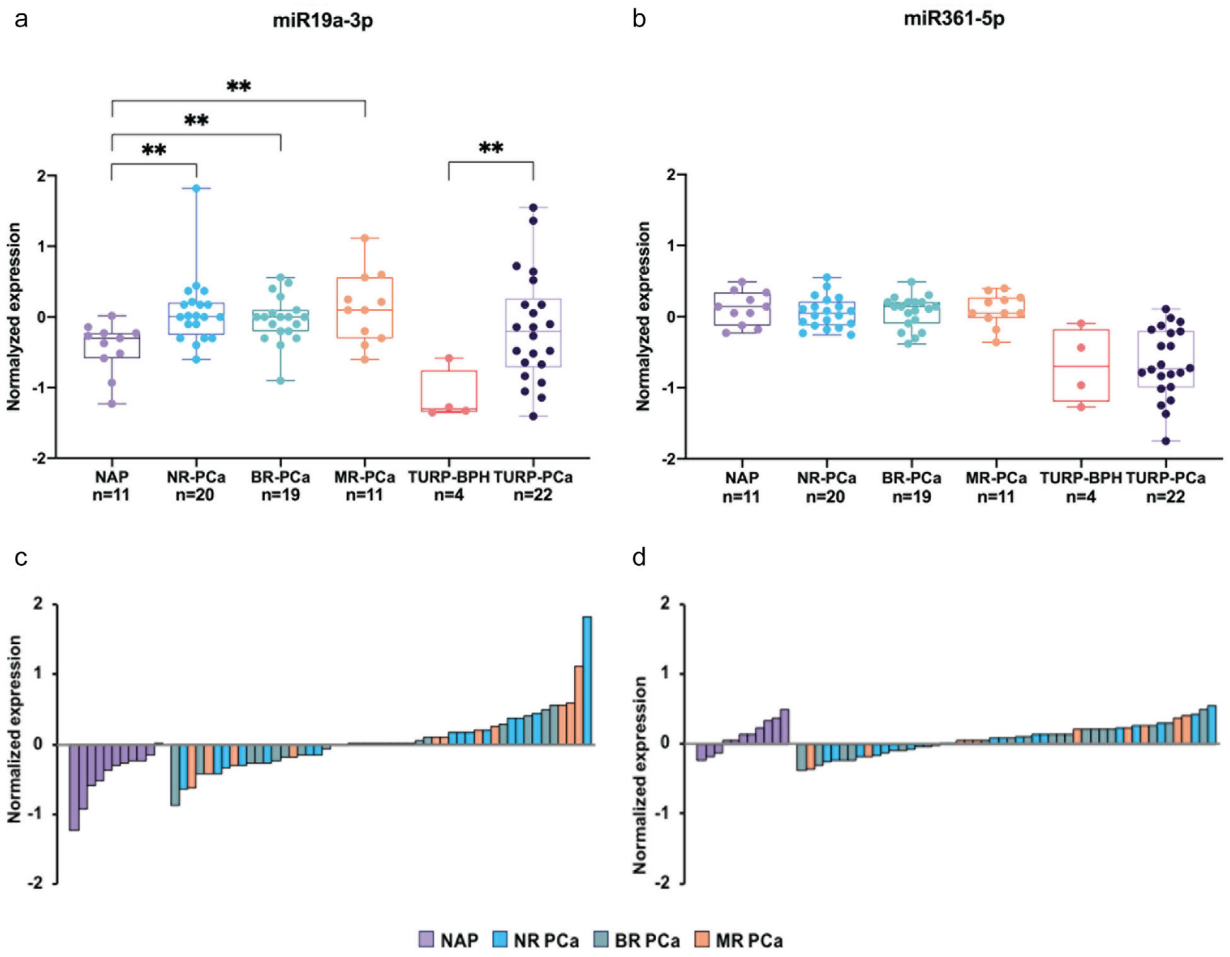
Expression of miR‐19a‐3p and miR‐361‐5p in clinical tissue samples. (a) and (b) Averaged expression of miR‐19a‐3p (a) and miR‐361‐5p (b) in normal adjacent prostate tissue (NAP), and organ‐confined prostate cancer tissue (PCa) from radical prostatectomy (RP) samples, benign prostate hyperplasia (BPH), and tumour issue derived by transurethral resection of the prostate (TURP‐BPH and TURP‐PCa). Patients presented with organ‐confined tumours at the time of RP and were stratified according to the clinical outcome at 15 years post RP; NR‐PCa, non‐recurrent cancer, BR‐PCa, biochemical recurrence after RP, MR‐PCa, macro‐metastatic recurrence after RP (Welch's t‐test, **P < 0.01). Normalised expression levels of mir‐19a‐3p (c) and miR‐361‐5p (d) in individual samples at the time of RP. The colour indicates clinical outcome after RP; abbreviations are as in (a) and (b).

To examine how the expression levels of miR‐19a‐3p and miR‐361‐5p relate to clinical samples, as plasma from a relevant cohort is currently unavailable, we investigated their expression in prostate cancer tissue. We interrogated the expression of both miRNAs in the Rotterdam prostate cancer patient archive cohort with 15 years clinical follow‐up comprising fresh frozen clinical tissue samples (Martens‐Uzunova et al., 2012). Samples in this cohort include normal adjacent (NAP, *n* = 11) and organ‐confined prostate tumour tissues (PCa, *n* = 50) obtained from radical prostatectomies (RP), as well as benign prostate hyperplasia (TURP‐BPH, *n* = 4) and advanced tumour tissues (TURP‐PCa, *n* = 22) obtained during TURP. MiR‐1307‐5p was excluded from the analysis as the microarray platform used for miRNA expression profiling of this cohort did not contain a probe set targeting miR‐1307‐5p. MiR‐19a‐3p was significantly upregulated in organ‐confined PCa (Welch's t‐test *P* value < 0.01) as well as in advanced TURP‐PCa (Welch's t‐test *P* value = 0.0035) compared to their normal counterpart tissues (Figure 8a).

A profound upregulation of miR‐19a‐3p was observed in advanced TURP‐PCa tissue. Accordingly, we evaluated the prognostic value of miR‐19a‐3p levels through stratification of patients from the organ‐confined PCa group based on prostate cancer recurrence after RP. However, no significant changes in the expression levels of miR‐19a‐3p at the time of RP were found between patients with non‐recurrent tumours and patients that presented with biochemical recurrence or developed metastatic disease after RP (Figure 8c). There was no significant change in the expression levels of miR‐361‐5p between normal and tumour tissue (Figure 8b, d), despite a lower expression of miR‐361‐5p in TURP samples, which originate from the transition zone of the prostate, compared to NAP and PCa tissues, which are generally located in the peripheral zone.

## 4 DISCUSSION

The EVs can mediate cancer metastasis, proliferation and survival as well as cancer drug resistance (Peinado et al., 2012; Soekmadji et al., 2016; Soekmadji et al., 2017; Wei et al., 2014). In prostate cancer, AR remains the best‐characterised driver of prostate cancer progression. Manipulation of the AR signalling axis alters the protein cargo in S‐EVs in LNCaP prostate cancer cells, and that the tetraspanin CD9 has been shown to be significantly more abundant in EVs from DHT‐treated cells when comparison with other S‐EVs markers including: TSG101, Alix, CD63 and CD81 (Soekmadji et al., 2016; Soekmadji et al., 2017; Soekmadji et al., 2017). Using interferometry‐based technology, SP‐IRIS to characterise in single EV level; we assessed the heterogeneity of circulating S‐EVs and the effect of androgens on CD9+ and CD63+ S‐EVs (Figures 2 and 3). The CD9+ S‐EVs are generally 5 nm larger (55 nm in diameter) than CD63+ and CD81+ S‐EVs, either in both plasma from CRPC patients (Figure 1) and those secreted by AR positive LNCaP and C42B cells (Figures 2 and 3). A theranostic antibody designed to specifically detect PSMA helps to further differentiate between circulating S‐EVs from prostate cancer patients, for both CD9 and CD63 captured S‐EVs (Figures 1c and 1d), providing enhanced potential to assess heterogeneity of S‐EVs for prostate cancer diagnosis. Androgens such as DHT and the clinical antagonist ENZ altered the size and profile of secreted vesicles (Figures 2 and 3). Interestingly, DHT treatment leads to increased cell surface area and the appearance of filopodia on the cell surface and expression of EV markers CD63 and CD9 on the cell surface of prostate cancer cells (Figure 4). Our cumulative data confirmed the role of androgens on S‐EV secretion in a single vesicle level.

Serum PSA levels are used to assess disease status and treatment response in metastatic prostate cancer. However, PSA is not always a reliable indicator of treatment effect in prostate cancer (Collette et al., 2005; Scher et al., 2007) and some patients have low PSA levels or non‐secretory tumours. Therefore, additional blood‐based biomarkers may aid prognostication, assess disease status, and measure treatment benefits in metastatic prostate cancer. Prior studies have suggested that miRNAs may be useful prostate cancer biomarkers (Bryant et al., 2012; Mitchell et al., 2008; Moltzahn et al., 2011; Sita‐Lumsden et al., 2013). In this study we demonstrate that S‐EVs and their cargo, which are secreted in response to treatment, could provide useful information on treatment response. Moreover, the heterogeneity of S‐EVs may provide a better strategy for prognostic biomarker, offering critical insights into disease biology and treatment resistance. There are reports showing the difference in drug response between AR expressing and non‐expressing prostate cancer cells. Prostate cancer cell lines co‐treated with doxorubicin or other drugs only partially target the multidrug resistance proteins in tumours (Rybalchenko et al., 2001), where a synergistic effect in AR negative PC3 and DU145 cells was observed, but not in the AR positive prostate cancer cells. This may suggest that an alternative pathway may be responsible for drug resistance in prostate cancer (Theyer et al., 1993), such as those regulated by EVs.

EVs can mediate drug resistance through the transfer of small RNAs, as was reported with EV derived miR‐221/222 in tamoxifen resistance of breast cancer cells (review in Soekmadji & Nelson, 2015). In prostate cancer, knockout of the DICER, a miRNA processing enzyme, resulted in LNCaP cells becoming unresponsive to androgen. Knock‐down of DICER also induced the expression of DICER corepressors, NCoR and SMRT (Narayanan et al., 2010), directly linking androgens to miRNA processing. For example, miR‐10a showed similar androgen regulation in LNCaP and VCaP cell lines and in xenograft models upon DHT treatment (Waltering et al., 2011). However, miR‐10a is also found in S‐EVs from AR negative PC3 (Hessvik et al., 2012), suggesting that the role of EV‐derived miR‐10a could be independent of androgens. Clinical data also support this observation, as shown in a study by Bryant et al. (2012). In that study, miR‐375 and miR‐141 were significantly increased in EVs from sera of metastatic patients compared with non‐recurrent prostate cancer patients.

The observed overexpression of snoRNA and tRNA fragments in DHT‐treated LNCaP cells in our study strongly resembles previous results showing upregulation of small RNAs associated with metastatic disease in clinical PCa specimens (Martens‐Uzunova et al., 2012; Martens‐Uzunova et al., 2015; Olvedy et al., 2016). In particular, the expression of snoRNA‐derived RNA sd78‐3p originating from the 3′‐end of SNORD78 and the tRNA‐fragment tRF‐LysCTT has been previously associated with recurrent metastatic disease (Martens‐Uzunova et al., 2015; Olvedy et al., 2016). Interestingly, while the overall small RNA composition of S‐EVs resembles that of the corresponding cells of origin, the response following DHT treatment of individual small RNAs between cells and S‐EVs is different. For example, snoRNAs demonstrate the strongest positive response upon DHT treatment in LNCaP cells, consistent with the increased demand for protein synthesis and ribosomal activity during androgen‐induced proliferation. Nevertheless, in S‐EVs, the levels of individual androgen‐responsive snoRNAs are decreased. Similarly, tRFs that demonstrate the strongest response to DHT treatment differ between cells and EVs.

Interestingly, we also observed several new mature miRNA forms that originated from the opposite arm of *bona fide* miRNA precursors. Whether this is a result of the differential sorting of specific small RNAs in EVs, or the formation of a different predominant type of EVs remains an interesting topic for future investigations. Nonetheless, this finding aligns with an increasing number of studies that suggest the existence of cellular mechanisms for selective packaging and release of miRNAs in EVs causing divergence in the intracellular and extracellular miRNA profiles (Arscott et al., 2013; Hessvik et al., 2012; Hessvik et al., 2013; Pigati et al., 2010; Wang et al., 2020).

It remains to be established if such sorting is for the clearance of small RNAs and miRNAs that are no longer needed and may otherwise have detrimental effects to the cell. This may be a consequence of these nucleic acids being disadvantageous for the cell. Alternatively, this may be a mechanism for ‘long distance’ cellular intervention. For example, EV miRNAs from tumour cells can be transported to other cells and influence the expression of target genes, causing changes in the tumour microenvironment response (Fabbri et al., 2012). It is also possible that both processes take place or are carried by different subpopulations of EVs. Therefore, identifying the biological function of small RNA EV cargo, for example, potential target genes of EV miRNAs, may provide important insights into the ongoing molecular process ongoing in cancer cells and their environment. In this study, we identified three miRNAs, miR‐19‐3p, miR‐361‐5p, and miR1307‐5p, with profoundly increased abundance in S‐EVs after androgen stimulation. Interestingly, downstream target gene analysis resulted in only one gene target, MDM4, common to all three miRNAs.

MDM4 (mouse double minute 4), is a nuclear protein and homolog to the negative regulator of p53, MDM2. MDM4 directly inhibits p53 transactivation function by binding its transcriptional activation and DNA binding domains in an MDM2 independent manner. MDM4 can also promote p53 protein synthesis under cellular stress by binding the nascent p53 transcript and altering its structure to facilitate MDM2 binding, which activates p53 translation via its internal ribosome entry site (IRES) (Chopra et al., 2017; Haupt et al., 2019; Lopez‐Pajares et al., 2008). These interactions are neatly regulated by various posttranslational modifications, including phosphorylation by several serine‐threonine kinases. For example, under growth‐promoting conditions, MDM4 is phosphorylated by AKT, a critical serine/threonine kinase in the PI3K pathway. Subsequent engagement of 14‐3‐3 increases MDM4–MDM2 multimerization, which in turn stabilises MDM2. Deregulation of this process poses a risk for pro‐survival AKT oncogenic activity (Lopez‐Pajares et al., 2008). Deregulation of the mTOR/AKT/PI3K pathway has been reported as one of the mechanisms underlying resistance to hormonal treatments (Crumbaker et al., 2017). Besides p53, MDM4 can also regulate the activity of AR by modulating its ubiquitination by MDM2. Chopra et al. (Chopra et al., 2017) reported that in LNCaP and 22RV1 cells, which express wild type AR, blocking MDM4 transcription by the small molecule inhibitor NCS207895 leads to reduced proliferation rates. Co‐overexpression of MDM2 and MDM4 are reported to stabilise AR protein by decreasing its ubiquitination. Still, this effect was only observed under low MDM4 dosage, suggesting that the ratio between both proteins is important for their regulatory function (Chopra et al., 2017).

In light of our results, an interesting question considering the regulation of MDM4 by miR‐19a‐3p, miR‐361‐5p and miR1307‐5p arises. It remains to be established in future studies if the three miRNAs are actively excreted from prostate cancer cells to prevent p53 stabilisation and subsequent inhibition of the androgen axis or if MDM4 transcript levels are actively decreased upon androgen stimulation to stabilise AR protein via modulating the stoichiometric ratio of the MDM2/MDM4 heterodimer. MDM2 and MDM4 are co‐amplified and co‐overexpressed in a large proportion of CRPC samples (Chopra et al., 2017) which pinpoints the need to better understand the role of S‐EVs in this process.

## CONFLICT OF INTEREST

The authors have declared no conflict of interest.

## Supporting information

Supporting information.

Supporting information.

Supporting information.

Supporting information.

Supporting information.
