# Supplementary material for: Androgens alter the heterogeneity of small extracellular vesicles and the small RNA cargo in prostate cancer
Source: J Extracell Vesicles. 2021 Aug 18;10(10):e12136. doi: 10.1002/jev2.12136 (PMC8374107; doi:10.1002/jev2.12136)

**Supplementary Figure 1. S-EV characterisation from C42B prostate cancer cells.** A) Treatment with androgens alters the diameter of S-EVs secreted by C42B cells; B) S-EVs were captured using CD9 or CD63 specific antibodies, and labelled by fluorescence tagged CD9, CD63 or PSMA antibodies. C42B conditioned medium was collected from three independent experiments. Data are mean ± SEM (*p<0.05). F: FBS, CSS: charcoal-stripped serum, DHT: dihydrotestosterone, ENZ: Enzalutamide





**Supplemental Figure 2.**

A representative Ponceau stained of blot performed on S-EVs and their parental cells under androgen treatments. LNCaP cells are cultured in 5% vesicle-depleted CSS with EtOH (vehicle, -DHT) or 10 nM dihydrotestosterone (DHT) with or without 10 µM Enzalutamide (ENZ) and cultured for further 48 h.


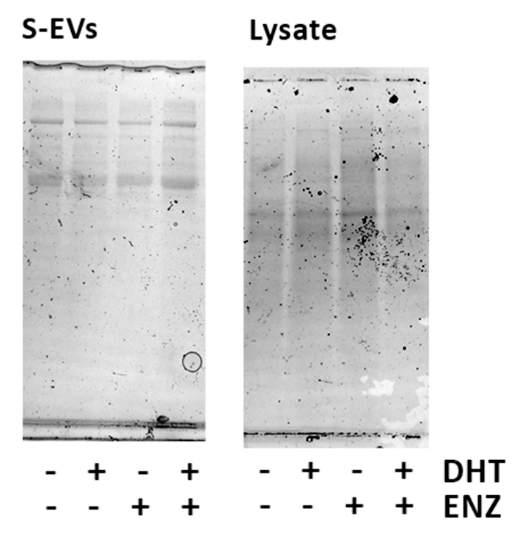

Supplement: Supplementary file 1 — Supporting information. [file JEV2-10-e12136-s003.docx]
